# Identifying the Stern-Gerlach force of classical electron dynamics

**DOI:** 10.1038/srep31624

**Published:** 2016-08-22

**Authors:** Meng Wen, Heiko Bauke, Christoph H. Keitel

**Affiliations:** 1 Max-Planck-Institut für Kernphysik, Saupfercheckweg 1, 69117 Heidelberg, Germany

## Abstract

Different classical theories are commonly applied in various branches of physics to describe the relativistic dynamics of electrons by coupled equations for the orbital motion and spin precession. Exemplarily, we benchmark the Frenkel model and the classical Foldy-Wouthuysen model with spin-dependent forces (Stern-Gerlach forces) to the quantum dynamics as predicted by the Dirac equation. Both classical theories can lead to different or even contradicting predictions how the Stern-Gerlach forces modify the electron’s orbital motion, when the electron moves in strong electromagnetic field configurations of emerging high-intensity laser facilities. In this way, one may evaluate the validity and identify the limits of these classical theories via a comparison with possible experiments to provide a proper description of spin-induced dynamics. Our results indicate that the Foldy-Wouthuysen model is qualitatively in better agreement with the Dirac theory than the widely used Frenkel model.

The electron couples to external electromagnetic fields via its charge as well as via its spin. Gradients of the electromagnetic fields induce a spin-dependent force in addition to the Lorentz force. Spin-dependent motion is implemented in the seminal Stern-Gerlach experiment^1^ and variants thereof^2^,^3^. Effects of spin-dependent forces appear in condensed matter^4^, in astrophysical systems^5^, in quantum plasmas^6^,^7^, and at relativistic electrons in strong electromagnetic fields^8^,^9^,^10^,^11^,^12^,^13^. A consistent theoretical framework for the description of particles with internal angular momentum is provided by the Dirac equation^14^. The application of this quantum-mechanical theory, however, is not always feasible and/or necessary if quantum effects are not important. Classical models of charged point-like particles with spin in electromagnetic fields are appealing because they are usually simpler from a mathematical point of view than the Dirac equation and are easier to interpret. A first covariant theory to describe the dynamics of a charged particle with spin was proposed by Frenkel in 1926 by purely classical considerations^15^. The Frenkel model has been employed in many studies and continues stimulating new research^16^,^17^,^18^,^19^,^20^,^21^. Considering that the spin was introduced as an intrinsic quantum feature of the electron^22^ it may, however, appear appropriate to start from quantum theory to find a classical model for the electron. Such a model can be derived from relativistic quantum theory by applying the correspondence principle to the Heisenberg equation for the time evolution of the position, the kinematic momentum, and the spin in the Foldy-Wouthuysen representations of the Dirac equation^23^,^24^. Both kinds of classical models are currently employed in different branches of physics, e. g., the classical Foldy-Wouthuysen model in gravitational fields^25^ or in crystals^26^, and the Frenkel model in astrophysics^5^ or in plasma fields^27^. Nevertheless, the validity and the limits of those classical models have not been studied, e. g., by a comparison to the Dirac theory. The lack of a widely accepted classical description of the electron is deeply related to interpretation problems regarding kinematic momentum operators in the Dirac theory^28^ and identifying an accurate classical model may also facilitate insights into quantum theory and will be valuable for systems where quantum descriptions are too complex such as for many-particle systems in extreme laser pulses.

## Results

The dynamics of a classical particle with rest mass *m*, charge *q*, and internal spin degree of freedom are governed by the modified Lorentz equation


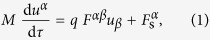


which incorporates the model-specific spin-dependent force 
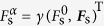
, and the Thomas-Bargmann-Michel-Telegdi equation^29^,^30^


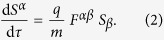


Here, *τ* denotes the proper time of the particle with d*τ* = d*t*/*γ*, *u*_*α*_ = d*r*_*α*_/d*τ* = (*γc*, −***p***/*m*)^Τ^ the four-velocity, *r*_*α*_ = (*ct*, −***r***)^Τ^ the time-space coordinate, 
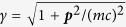
 the relativistic Lorentz factor, *c* the speed of light, *F*^*αβ*^ the electrodynamic field tensor, ***p*** the kinematic momentum, *M* the effective mass, *S*^*α*^ = (*S*^0^, ***S***)^Τ^ the spin’s four-vector in the laboratory frame with


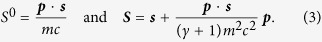


The classical spin vector in the rest frame ***s*** of length *ħ*/2 is proportional to the particle’s polarisation and corresponds to the spin operator in quantum mechanics. The spin-dependent forces may be written for the classical Foldy-Wouthuysen model (FW) and the Frenkel model (F) as^15^,^23^


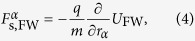



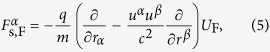


with the scalars *U*_FW_ and *U*_F_ defined as


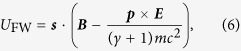



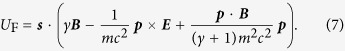


The effective masses in these two models are *M*_FW_ = *m* and *M*_F_ = *m* − *qγU*_F_/(*mc*^2^), respectively. The forces (4) and (5) become equal in the limit of low electron energies. The classical Foldy-Wouthuysen and the Frenkel models differ mainly in the large-kinematic-momentum limit.

The spin-dependent forces (4) and (5) are gradient forces, which become large in systems of ultra strong laser fields in the short-wavelength limit. In the following, we consider the interaction of relativistic electrons with strong electromagnetic fields in the X-ray regime. The electron moves initially in *x* direction opposite to the plane-wave laser pulse, which is assumed to have linear polarisation in *y* direction and to be modulated by a sin^2^-shaped envelope. At time zero, the front of the laser pulse reaches the origin of the coordinate system, where the electron is initially located. The electron’s initial spin orientation is parallel or anti-parallel to the direction of the magnetic field (*z* direction), representing spin up (indicated by ↑) or down (indicated by ↓) states. Note that as a consequence of equation (2) the spin remains in its initial state for all times for the considered setup.

**Figure 1:**
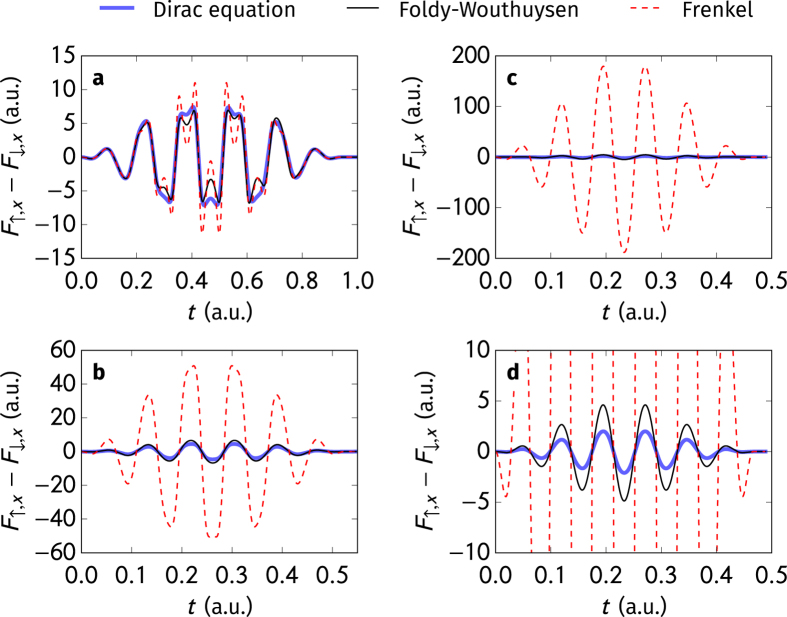
Difference between the force components in laser propagation direction for electrons in a strong plane-wave laser pulse with initial spin orientation parallel or anti-parallel to the magnetic field direction as predicted by the various considered models. The four sub-figures correspond to different initial electron momenta opposite to the propagation direction of the laser pulse. (**a**) ***p*** = (0, 0, 0)^Τ^; (**b**) ***p*** = (−*mc*, 0, 0)^Τ^; (**c**,d) ***p*** = (−2*mc*, 0, 0)^Τ^. Sub-figures (**c**,**d**) show the same data but on different scales. Laser parameters are peak electric field strength 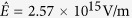, wavelengths *λ* = 1.06 nm, the pulse length equals *n* = 6 cycles. In case of the Dirac equation the wavepacket had an initial width of 0.026 nm.

Due to the spin-dependent forces, the electron’s trajectory depends on the spin orientation. Although the influence of the spin on the shape of the trajectory is very small, the spin-dependent force may be used to compare the three models. In the classical models the total force can be split directly into a Lorentz force part and a spin force part (see equation (1)), which is not possible in the framework of the Dirac equation. Therefore, the magnitude of the spin-dependent force is evaluated by calculating the difference of the total forces ***F***_↑_ and ***F***_↓_ for the trajectories of electrons with initial spin parallel and anti-parallel to the *z* direction. Figure 1 shows the *x* component of ***F***_↑_ − ***F***_↓_ as a function of time *t* as determined from the Dirac equation, the classical Foldy-Wouthuysen model, and the Frenkel model. For zero initial momentum, the three models yield very similar results, as shown in Fig. 1(a). In particular, the predictions of the Dirac equation and the classical Foldy-Wouthuysen model for the force difference *F*_↑,*x*_ − *F*_↓,*x*_ match very well and the prediction of the Frenkel model shows only small deviations from the other two models. The qualitative predictions of the various models diverge with growing initial electron momentum, see Fig. 1(b–d). The Frenkel model predicts that the force difference becomes larger for relativistic electrons, while the Dirac equation and the classical Foldy-Wouthuysen model yield smaller force differences. The qualitatively different behaviour of the Frenkel model and the classical Foldy-Wouthuysen model is a consequence of a different dependence on *γ* of the spin-dependent forces (4) and (5).

For symmetry reasons the net effect of the plane-wave pulse on the electron momentum vanishes in the Frenkel model as well as the classical Foldy-Wouthuysen model, although both models predict different forces acting on the electron during its interaction with the laser pulse. Therefore, a plane-wave setup is not suitable to test the classical models experimentally. However, considering focused infrared laser pulses of upcoming high-power laser facilities the discrepancy in the predicted electron dynamics by the classical Foldy-Wouthuysen and the Frenkel models becomes large enough to distinguish between them experimentally.

An electron, which is initially directed towards the focus of a counter-propagating high-intensity laser pulse with linear polarisation, is displaced transversely due to the transverse electric field. When the oscillating field changes its sign, the force drives the electron back to its initial transverse position. However, this force is smaller than the expelling force due to the focusing inhomogeneity. As a result, the oscillation centre of a spinless charged particle drifts radially from the spot centre, which is called ponderomotive scattering^31^. Beside the deflection of a charged particle in the ponderomotive potential of the laser fields, the spin may induce a further deflection via the spin-dependent forces (4) and (5), in particular, if the electron is polarised parallel or anti-parallel to the direction of the magnetic field. As the spin is (anti-)parallel to the magnetic field direction it follows from the equations (1) and (2) that the electron remains in the plane perpendicular to the magnetic field direction and the electron’s spin is frozen to its initial state. The deflection of a particle in the ponderomotive potential of the focused laser pulse is defined by the angle *θ* between its initial momentum and its final momentum after the particle is separated from the laser fields. It is dominated by the ponderomotive scattering due to the dominant Lorentz force, which increases with increasing field strength.

**Figure 2:**
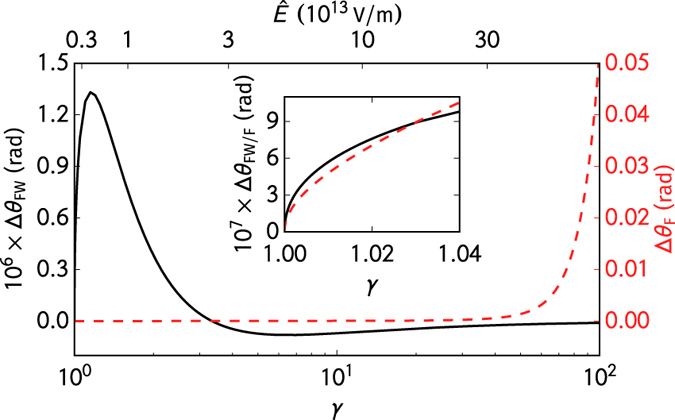
The aberration angle Δ*θ* between spin-up and spin-down electrons induced by the ponderomotive potential as a function of the initial energy *γmc*^2^ of the particle for the classical Foldy-Wouthuysen (solid black line, left scale) and Frenkel (dashed light red line, right scale) models. The inset shows the non-relativistic limit. The electric field strength of the counter-propagating laser pulse scales with initial *γ* as 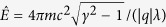, which causes a strong acceleration of the electron opposite to its initial velocity but without reflecting it. Other parameters are the wavelength *λ* = 800 nm, the duration (number of cycles) *n* = 20, and focus radius *w*_0_ = 2*λ*.

Besides the deflection due to the charge, the electron’s spin state leads to a modification of the deflection angle *θ*, which depends on the spin orientation. In this way, one can define the aberration angle Δ*θ* = *θ*_↑_ − *θ*_↓_, where *θ*_↑_ and *θ*_↓_ denote the deflection angles for the spin-up and spin-down cases, see also Methods section. The Frenkel and the classical Foldy-Wouthuysen models lead to different aberrations Δ*θ*, as shown in Fig. 2 for varying electron energies. As indicated in the inset, the two models share the same non-relativistic limit. In the relativistic regime, the angle Δ*θ* as predicted by the classical Foldy-Wouthuysen model does not vary with the electron’s initial energy monotonically and it may even change its sign. Furthermore, the absolute value of the spin-induced additional deflection angle Δ*θ*_FW_ from the classical Foldy-Wouthuysen model remains under the magnitude of 10^−6^ rad and decreases with the electron’s initial energy in the relativistic parameter regime. In contrast to the classical Foldy-Wouthuysen model, the aberration angle of the Frenkel model Δ*θ*_F_ increases to about 0.05 rad with the electron’s energy for relativistic electrons in high-intensity laser fields of the applied parameters.

## Discussion

We have investigated the dynamics of electrons in various setups by applying two different classical models, the classical Foldy-Wouthuysen and the Frenkel models. The predictions of these classical models were compared to each other and to predictions by the Dirac equation, when a numerical solution of the Dirac equation was feasible. In specific parameter regimes, these classical models can lead to conflicting predictions. The Frenkel model may be of timely interest^5^,^27^,^32^ and prominent^33^,^34^ for its much longer history and its wide application^35^,^36^. The classical Foldy-Wouthuysen model, however, may be superior as it is qualitatively in better agreement with the quantum mechanical Dirac equation.

The discrepancies in the predictions of the two classical models may become experimentally detectable in light-matter interaction in strong highly focused beams. As electron bunches with the emittance as low as 10^−3^ rad have been prepared^37^, the spin-induced aberration angle of the order of 10^−2^ rad from the Frenkel model is potentially measurable, if an electron beam with an energy of tens of MeV and an infrared laser of the intensity ~10^22^ W/cm^2^ are applied as discussed above. The spin-induced contribution to the deflection as predicted by the classical Foldy-Wouthuysen model, which is for the applied parameters of the order of 10^−6^ rad, is too small to be demonstrated. However, a differentiation among both predictions appears feasible. In current head-on experiments^38^,^39^ with focused fields of high inhomogeneities and energetic electrons no significant spin effect in orbital motion was observed. The lack of experimental evidence for a non-negligible spin-induced deflection may be seen as a superiority of the classical Foldy-Wouthuysen model again regarding spin modified dynamics.

## Methods

For the plane-wave setup, the laser pulse is assumed to have linear polarisation in the *y* direction and is modulated by a sin^2^-shaped envelope


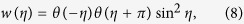


where *θ* (*η*) denotes the Heaviside step function. Introducing the wavelength *λ*, the peak amplitude 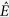, and the pulse width *n* measured in laser cycles, the electric field component of the laser pulse is given by


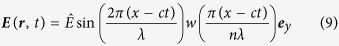


and the magnetic field component follows via ***B***(***r***, *t*) = ***e***_*x*_ × ***E***(***r***, *t*)/*c*. At time zero, the front of the laser pulse reaches the origin of the coordinate system, where the electron is initially located. The electron’s initial spin orientation is parallel or anti-parallel to the direction of the magnetic field (*z* direction). We solved the equations of motion of the classical Foldy-Wouthuysen model and the Frenkel model for the plane-wave setup numerically via the Boris algorithm^40^. The time-dependent Dirac equation for a two-dimensional wavepacket in the same setup was propagated numerically employing a Fourier split operator method^41^,^42^,^43^,^44^. In order not to violate the quantum-classical correspondence between classical operators and quantum mechanical operators, the Dirac wavepacket was prepared to have a small width compared to the wavelength of the applied electromagnetic field^45^. The force, which acts on the electron during its interaction with the plane-wave electromagnetic field and which enters in Fig. 1, is given by equations (4) and (5). In the case of the Dirac equation, the force was determined as the time derivative of the quantum mechanical expectation value of the electron’s kinematic momentum 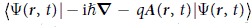, where Ψ(***r***, *t*) is the electron’s four-component wave function and ***A***(***r***, *t*) denotes the vector potential of the electromagnetic fields.

For the setup with a focused infrared laser pulse, numerical solutions of the Dirac equation are not feasible due to the long time scale of infrared laser pulses. A longer pulse length in combination with wavepacket spreading leads to a *λ*^3^- or *λ*^4^-scaling of the computational demand to solve the Dirac equation in two or respectively three dimensions. Thus numerical simulations were limited to the two classical models.

**Figure 3:**
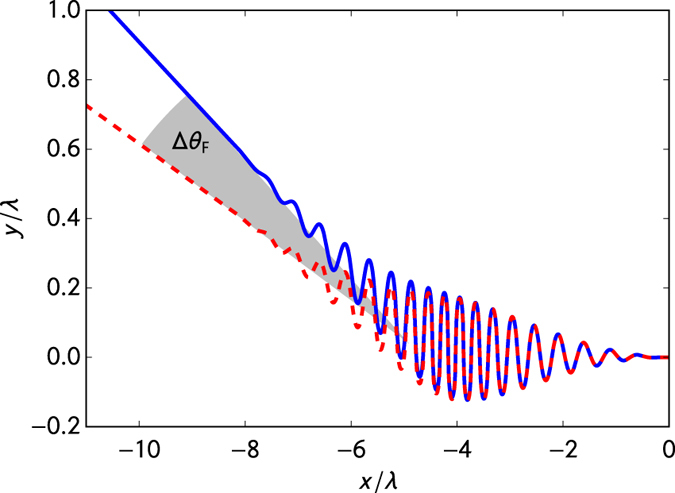
Definition of the aberration angle Δ*θ*. Lines represent trajectories of a highly energetic electron with initial *γ* = 100 in a focused laser pulse with the wavelength *λ* = 800 nm, the amplitude of strength 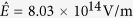, the duration (number of cycles) *n* = 20, and focus radius *w*_0_ = 2*λ*. The solid and dashed curves correspond to those of electrons with the spin parallel to the *z* axis and anti-parallel to the *z* axis (magnetic field direction), respectively, as described by the Frenkel model. The deflection angle for the Frenkel model is indicated by Δ*θ*_F_.

The polarisation and the longitudinal (in propagation direction) profile of the focused laser pulse are as in the plane-wave case. The transverse profile and the phase are modelled as a Gaussian beam with the transversal focus radius *w*_0_, e.g. with terms up to the 5th order of the small diffraction angle *ϵ* = *w*_0_/*x*_*r*_ as in ref. 46, where 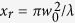 is the Rayleigh length. The phase of the focused pulse depends not only on the longitudinal coordinate but also on the transverse coordinate. The deflection of an electron in a head-on collision with a focused laser pulse is defined by the angle between the final transverse and the longitudinal momentum components after the particle is separated from the laser fields. Due to spin-dependent forces the deflection depends on the electron’s initial spin orientation (relative to the magnetic field direction). In this way, the aberration angle Δ*θ* is defined as the angle between the final momenta for electrons with initial spin-up and spin-down orientation, see Fig. 3.

The considered setup with the employed parameters is also sensitive to radiative reaction forces. Our calculations involving both spin and radiative reaction forces (via the Landau-Lifshitz equation^47^) have, however, confirmed that the key deviations displayed in Fig. 2 are not essentially modified such as especially the strong rise of the aberration angle Δ*θ* for the Frenkel model as compared to the Foldy-Wouthuysen model in the highly relativistic regime. Because of this and since the Landau-Lifshitz equation has not been confirmed experimentally as well, we decided here to present comparisons not including the radiative reaction forces.

## Additional Information

**How to cite this article**: Wen, M. *et al*. Identifying the Stern-Gerlach force of classical electron dynamics. *Sci. Rep.* **6**, 31624; doi: 10.1038/srep31624 (2016).
